# Clinical features and pitfalls in the laboratory diagnosis of dengue in travellers

**DOI:** 10.1186/1471-2334-6-120

**Published:** 2006-07-21

**Authors:** Ole Wichmann, Klaus Stark, Pei-Yun Shu, Matthias Niedrig, Christina Frank, Jyh-Hsiung Huang, Tomas Jelinek

**Affiliations:** 1 Berlin Institute of Tropical Medicine, Spandauer Damm 130, 14050 Berlin, Germany; 2 Robert Koch Institute, Seestrasse 10, 13353 Berlin, Germany; 3 Center for Research and Diagnostics, Center for Disease Control, Department of Health, 161 Kun-Yang Street, Taipei 115, Taiwan, Republic of China

## Abstract

**Background:**

Several enzyme-linked immunosorbent assay (ELISA)-kits are commercially available for the rapid diagnosis of dengue infection, and have demonstrated good sensitivity and specificity in paired serum samples. In practice, however, often only one blood sample is available from febrile travellers returning from dengue endemic areas.

**Methods:**

To evaluate the diagnostic value of positive dengue antibody-titres performed by a standard ELISA (PanBio IgM- and IgG-ELISA) in single serum samples (regarded as "probable infection"), 127 positive samples were further analyzed using envelope/membrane IgM-, and nonstructural protein 1 IgM- and IgG-ELISAs, immunofluorescence assays, and real-time reverse transcription polymerase chain reaction assays (RT-PCR). A combination of the test-results served as the diagnostic "gold standard". A total of 1,035 febrile travellers returning from dengue-endemic countries with negative dengue-serology and RT-PCR served as controls to compare clinical and haematological features.

**Results:**

Overall, only 64 (positive predictive value = 50%) of the probable cases were confirmed by additional analysis and 54 (42.5%) were confirmed to be "false-positive". Rash was the only clinical feature significantly associated with confirmed dengue fever. The combination of thrombocytopenia and leucopenia was present in 40.4% of confirmed and in 6.1% of false-positive cases. Thus, the positive predictive value for the combination of positive PanBio-ELISA plus the two haematological features was 90.5%.

**Conclusion:**

The examination of paired serum samples is considered the most reliable serodiagnostic procedure for dengue. However, if only one blood sample is available, a single positive ELISA-result carries a high rate of false-positivity and should be confirmed using a second and more specific diagnostic technique. In the absence of further testing, platelet and white blood cell counts are helpful for the correct interpretation.

## Background

The increase in international air travel, and the increasing transmission of dengue in the tropics mean that health care providers in western countries are more likely to be confronted with travel-acquired dengue infections [1]. Worldwide, dengue is nowadays regarded as the most important arboviral disease of humans and is endemic in more than 100 tropical and subtropical countries [2].

Clinically, most dengue infections in international travellers present either asymptomatically or as a febrile illness, often accompanied by headache and severe myalgia [3-5]. Currently three basic diagnostic methods are used in laboratories, including viral isolation, detection of the genomic sequence by a nucleic acid amplification technology assay, and detection of virus-specific antibodies [6]. However, due to labour intensiveness and high costs, the first and the second method are rarely available even in specialized travel clinics.

The serological diagnosis of dengue is limited by the fact that antibodies usually arise in the late stage of the acute illness. Several studies have demonstrated that only in a small proportion of infected patients, immunoglobulin M (IgM) is detectable during the first 3 to 4 days after onset of symptoms [7,8]. Cross reactivity with several other flaviviruses and previous immunizations in travellers against yellow fever (YF), Japanese encephalitis (JE), and tick-borne encephalitis may pose additional problems in the interpretation of the serological results [9]. Thus, the examination of paired serum samples is considered the most reliable serodiagnostic procedure with increasing titres being required to confirm the diagnosis. In contrast, the detection of dengue IgM-antibodies only indicates "probable" infection [10].

In clinical practice, however, frequently only a single serum sample is available from a febrile traveller who returned from the tropics. In patients seeking medical care for post-travel illness, malaria needs to be excluded in a first step as it might be fatal if not treated as soon as possible. Since dengue is the most common arboviral disease in man, a second diagnostic step would be the interpretation of a dengue serological result from the first serum sample.

Several enzyme-linked immunosorbent assay (ELISA)-kits including dipstick systems are commercially available for the rapid diagnoses of dengue virus infection and demonstrated good sensitivity and specificity in paired serum samples [11-13]. However, most of those tests have been evaluated with paired sera of patients living in endemic area, most often in Asia. For these reasons, a study has been performed to evaluate the diagnostic value of a positive single specimen test result revealed by a standard ELISA-kit (PanBio-IgM- and IgG-ELISA) in ill travellers returning from dengue endemic countries in different parts of the world and how to increase its predictive value with the help of simple clinical and haematological features. Data regarding the geographical risk and the development of dengue antibody-prevalence over the study-period have been published elsewhere [14].

## Methods

### Sampling

Blood samples were collected at the Berlin Institute of Tropical Medicine, Germany. Approximately 5,000 patients annually seek medical assistance post travel at the outpatient department (OPD) of the institute. Before consultation each patient is asked to fill out a questionnaire to provide information on major complaints, travel destination, travel duration, risk behaviour and preventive measures before and during the journey.

Patients were included in this study who presented during the time periods 1996 to 1998 and 2002 to 2004 at the OPD with onset of fever during travel in dengue endemic areas or up to 12 days post return. Available banked sera, initially obtained for diagnostic purposes, were retrospectively investigated for acute or recent dengue infection. To compare patients with and without dengue infection and to take into account "non-febrile" presentations of the infection, clinical and laboratory data of travellers returning from same destinations but with complaints other than fever were collected. For convenience, patients were included in this group who were suffering from non-febrile diarrhoea. All data were retrieved from the patients' medical charts and the above mentioned questionnaires. The research has been conducted in accordance to the Central Ethics Committee of the German Federal Medical Association and in compliance with the Helsinki Declaration.

### Laboratory testing

#### Screening tests

All serum samples of included patients were screened for dengue antibodies by using both a commercially available IgM-capture ELISA and an IgG indirect ELISA (PanBio Pty Ltd., East Brisbane, Australia). A probable acute infection was defined according to the manufacturer's instruction as having a sample:calibrator absorbance ratio of IgM ≥ 1.0 (defined as a titre of ≥ 10 U) and/or (characterizing secondary infections) of IgG ≥ 4.0 (defined as a titre of ≥ 40 U).

#### Confirmation of positive screening test results

**Figure 1:**
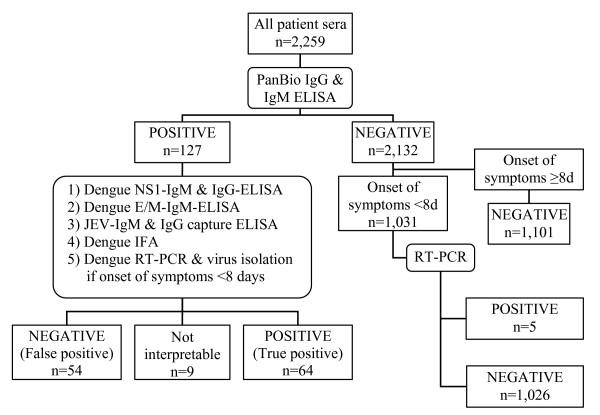
**Laboratory tests performed within the study population**. IFA = immunofluorescence assay. JEV = Japanese encephalitis virus. NS1 = non-structural protein 1. E/M = envelope/membrane. RT-PCR = real-time reverse transcription polymerase chain reaction.

As outlined in figure 1, all serum samples with positive ELISA results were further investigated to confirm the diagnosis by using envelope/membrane (E/M) and nonstructural protein (NS) 1 serotype-specific capture IgM ELISAs and a NS1 serotype-specific IgG ELISA as previously described: Briefly, for the E/M serotype-specific capture IgM ELISA, microliter wells were incubated with 100 μL of a cocktail containing 1 μg of monoclonal antibody D56-3 per mL and diluted culture supernatant of DEN-1, DEN-2, DEN-3, DEN-4, or JE virus-infected Vero cells [15]. The assay of NS1 serotype-specific capture IgM ELISA is similar to that of E/M serotype-specific capture IgM ELISA with two differences: Monoclonal antibody D2/8-1 was used in the NS1 capture IgM ELISA, and patient serum samples were used at a 1:20 dilution. The wells for the NS1 serotype-specific IgG ELISA were incubated with 1:3-diluted NS1-containing culture supernatants of DEN-1, DEN-2, DEN-3, DEN-4, or JE virus-infected Vero cells in PBST-1% BSA-5% normal rabbit serum [16]. Furthermore, an additional testing was performed by an immunofluorescence assay (IFA) (Euroimmun AG, Luebeck, Germany).

By definition, a screening test result was classified as "true positive" if the combined analysis of all four tests was positive. If the results of these four confirmatory antibody tests were discrepant, sera collected during the acute phase of illness were processed using real-time reverse transcription polymerase chain reaction assays (RT-PCR) for the detection of viral nucleic acid as described elsewhere [17] combined with virus isolation in cell culture. A positive RT-PCR or virus isolation confirmed the dengue infection, regardless of discrepant confirmatory tests. Discrepant confirmatory test results in combination with a positive screening test were classified as "not interpretable", also if RT-PCR or virus isolation were negative.

#### Analysis of negative screening test results

If sera tested negative with the initial PanBio-ELISA, all blood samples that were collected during the acute phase of illness (i.e. time from onset of illness until blood collection less than 8 days) were further investigated by using RT-PCR. For this purpose, included sera were processed in pools of 5 samples. In a second step, each sample of a positive pool was individually tested by RT-PCR.

#### Haematological parameter

Leucopenia was defined as a white cell blood count below or equal to 4/nL and thrombocytopenia was defined as a thrombocyte count below or equal to 150/nL.

### Statistical methods

All data were entered into a Microsoft Access database (Microsoft Access 2002, Microsoft Corp., Redmond, WA) and exported to SPSS (version 13.0 for Windows; SPSS Inc., Chicago, IL) for statistical analyses. Descriptive statistics were used to describe the frequencies of patients' symptoms and the laboratory investigations. Odds ratios and 95% confidence intervals (CI) were determined for the comparisons of clinical and laboratory features of travellers with and without dengue infections. As the haematological and serological data were not normally distributed, the median values were calculated, and when comparing two independent variables the Mann-Whitney rank sum test was used. A statistically significant difference was determined by a p-value <0.05.

## Results

A total 2,259 patients with a median age of 33 years (range 2 – 79) were eligible to be included in the study. Of these 1,163 were male and 1,096 were female. Reasons for presenting at the OPD were either travel-associated fever (n = 1,091) or diarrhoea without fever (n = 1,168). The patients were classified according to the time of blood collection with 35% presenting 1 to 3 days, 20% presenting 4 to 6 days, 12% presenting 7 to 9 days, and 33% presenting 10 days or later after the onset of symptoms. A total of 1,020 patients had recently returned from Asia (n = 514 with fever; n = 506 with diarrhoea), 685 returned from Africa (n = 345 with fever; n = 340 with diarrhoea), and 550 from South-Central America and the Caribbean (n = 230 with fever; n = 320 with diarrhoea). Four patients travelled to more than one continent during their trip (n = 2 with fever; n = 2 with diarrhoea).

By screening all patients' sera with the Pan-Bio IgM- and IgG-ELISAs, results indicated a probable dengue infection in 127 (5.6%) cases (n = 89 or 8.2% in patients with fever; n = 38 or 3.3% in patients with diarrhoea). Of these, 10 were positive defined by high IgG-antibody-titres only, and 4 had both high IgG- and IgM-antibody titres.

As described in the method section, the decision of whether a screening test result was classified as true positive or as false positive has been established with the combination of four confirmatory antibody tests (E/M-specific and NS1 serotype-specific capture IgM ELISAs, NS1 serotype-specific IgG ELISA, and IFA). Performed on each of the 127 samples, the results of these four confirmatory tests were concordant in 115 samples (61 concordant positive, and 54 concordant negative), and discrepant in 12 samples. Among these 12 samples with discrepant confirmatory antibody tests, three were positive in RT-PCR or virus isolation. In summary, the additional analysis of the 127 sera of patients with probable dengue infection (i.e. positive PanBio ELISA) confirmed the disease in 64 (positive predictive value = 50%). Fifty-four (42.5%) samples were classified not to be dengue fever (false positive sera). For 9 (7%) samples results were classified as not interpretable due to discrepant results of the confirmatory tests. The serotyping-results of the confirmed infections have been published previously [14].

**Figure 2:**
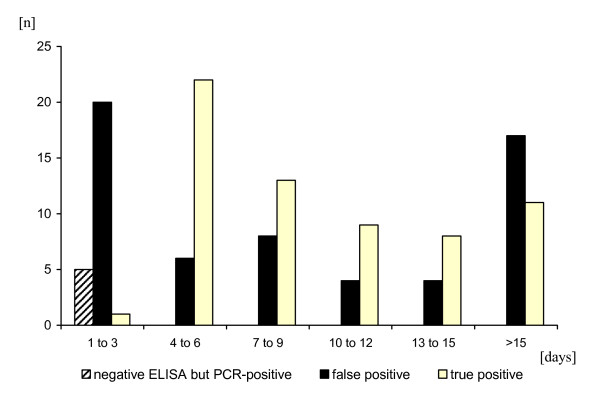
**Test interpretation of combined PanBio-IgG and IgM ELISA**. Results are presented according to time of blood collection (days after onset of illness). A combination of four tests (E/M-specific and NS1 serotype-specific capture IgM ELISAs, NS1 serotype-specific IgG ELISA, and IFA) were used to classify the PanBio-ELSIA test result as true positive and false positive.

A total of 1,031 PanBio ELISA-negative sera collected during the acute phase of illness were further investigated by RT-PCR. Of these, 5 turned out to be viraemic yielding a total of 69 confirmed dengue-infections. ELISA-negative but RT-PCR positive cases were observed only during the first three days of symptoms but not on day 4 to 7 (Figure 2).

### Details of serological results

Of the 10 probable cases with high positive IgG-antibody levels only, 5 turned out to be true positive and 4 turned out to be false positive (IgG titres were 44 U, 47 U, 69 U, and 94 U on day 2, 5, 35, and 36, respectively). In one case the confirmatory tests were discrepant. All 4 samples with both positive IgG and IgM antibodies in the PanBio ELISA were classified as true positive in accordance with the four confirmatory tests.

A higher frequency of false positive PanBio-ELISA results was observed in the first three days after onset of symptoms, and in cases where the blood collection was performed more than 15 days after the onset of symptoms (Figure 2).

Travelling in Southeast-Asia was associated with a significantly lower risk of the PanBio-ELISA showing a false positive result compared to other destinations (p-value = 0.0001). In patients returning from Sub-Saharan Africa 10 of 15 (67%) samples, returning from the Indian subcontinent 16 of 25 (64%) samples, and returning from Latin-America and the Caribbean 15 of 27 (55.6%) samples turned out to be false positive. In contrast, only 13 of 51 (25.5%) samples from patients travelling in Southeast-Asia had false positive results.

In false positive sera, the median IgM-titre was significantly lower than in confirmed cases' sera (13 vs. 29.5 U, respectively, p < 0.001). However, 8 samples with false positive results revealed an IgM-titre higher than 20 U (maximum 70). One serum of a febrile traveller returning from Thailand (PanBio-ELISA results: IgM 18 U and IgG negative on day 8 after onset of fever) was found to be positive for JEV-antibodies (IgM and IgG positive with a JEV-capture ELISA) which was confirmed by a JEV-specific NS1 IgG ELISA.

### Clinical and laboratory features

Table 1 summarizes the clinical features of 69 travellers with confirmed dengue infection in comparison to 1,035 febrile travellers without dengue infection. In addition to fever, main clinical features of dengue were headache (62%) and muscle pain (50%). When comparing patients with and without dengue-infection, there was no significant difference in the frequencies of major symptoms except in the occurrence of a skin rash (23.5% vs. 9%, respectively, p = 0.0001).

**Table 1:** Clinical symptoms of studied travellers. Travellers with confirmed dengue virus infection are compared with febrile travellers without dengue (negative dengue serology and negative dengue RT-PCR).

	Travellers with confirmed dengue infection (n = 69)	Febrile travellers without dengue (n = 1,035)	OR (95% CI) confirmed vs. fever controls
Fever (%)	56/69 (81.2)	1,035/1,035 (100)	**-**
Headache (%)	41/66 (62.1)	460/941 (48.9)	1.7 (1.0–3.0)
Muscle pain (%)	33/66 (50.0)	354/940 (37.7)	1.7 (0.98–2.8)
Diarrhoea (%)	30/69 (43.5)	397/1,035 (38.4)	1.2 (0.7–2.1)
Nausea (%)	15/67 (22.4)	189/944 (20.0)	1.2 (0.6–2.2)
Skin rash (%)	16/68 (23.5)	85/948 (9.0)	**3.1 (1.6–5.9)**

A total of 13 patients (19%) with confirmed dengue-virus infection were afebrile on the day of presentation and denied a history of recent fever. Of 6 travellers acquiring dengue in Africa, 4 (66.7%) had no history of fever. This rate was significantly lower in other geographic regions: 7 of 50 (14%) dengue-patients returning from Asia and 2 of 13 (15%) returning from the Americas and the Caribbean remained afebrile (p-value 0.01 and 0.046, respectively). Afebrile dengue-patients stated significantly lower frequencies of headache (23%) and myalgia (15%) (p = 0.001 and 0.004, respectively). Other symptoms among these patients were nausea (15%), skin rash (8%), and diarrhoea (100%). However, the latter symptom was used as an inclusion criterion to recruit afebrile travellers presenting at the OPD. Three of the 13 afebrile dengue patients also had pathogens identified in their stool samples (in two cases *Giardia lamblia *and in one case *Shigella*). In the remaining 10 patients, stool examinations were negative.

Haematological features of dengue included leucopenia und thrombocytopenia during the acute phase of illness (53.2% and 48.9%, respectively). The combination of leucopenia and thrombocytopenia was present in 40.4% of the confirmed, in 6.1% of the false positive (OR 10.5; 95% CI 2.1 – 72.0), and in 3.1% of the sero-negative fever-cases (OR 20.3; 95% CI 9.4 – 43.8). AST was increased in 5 of 26 (19.2%) dengue cases (4 mildly with levels not higher than 100 U/L and one patient higher than 100 U/L), and ALT was increased in 12 of 43 (27.9%) cases (3 patients with levels higher than 100 U/L). Lactate dehydrogenase was elevated in 7 of 12 dengue patients (58.3%) and in 18 of 142 (12.7%) sero-negative fever-cases (OR 9.6; 95% CI 2.4 – 40.1).

The overall positive predictive value of the PanBio ELISAs (IgM plus IgG) was 50%. The combination of a positive PanBio-ELISA result plus the presence of thrombocytopenia or plus both leucopenia and thrombocytopenia increased the positive predictive value of the test to 88.5% and 90.5%, respectively.

## Discussion

A total of 2,259 serum samples of travellers returning from dengue endemic countries were screened for the prevalence of dengue-antibodies and further analyzed to evaluate the diagnostic value of positive dengue antibody titres (IgM and IgG) in single serum samples. Clinical and haematological parameters of these patients were included in the analysis for their potential support of the diagnosis. The study was carried out at a large travel clinic in Europe, where the spectrum of travel-associated diseases might be suggested to be similar to those of other clinics in European countries that manage patients for post-travel illness.

The screening was performed with two commercially available standard ELISAs (PanBio indirect IgG ELISA and IgM capture ELISA), which are frequently used in clinical settings worldwide. The combination of these two tests showed excellent sensitivity (99–100%) in populations of endemic countries in Asia [13,18]. Vaughn et al. demonstrated a high specificity (92%) in paired sera from Thai patients without flavivirus infection, although 45% of patients with JE showed elevation of IgG but not IgM [13].

Only few studies have been performed in travellers to evaluate the specificity and sensitivity of these tests, even though it might be expected that in this particular population other pathogens and previous immunizations create a potential problem of cross reactivity, especially if more specific but also more costly test such as RT-PCR or plaque reduction neutralization test (PRNT) are not available. Schwartz et al. were able to demonstrate that among 82 JE and/or YF vaccinated Israeli travellers the IgM test was negative in all healthy vaccines, and thus highly specific. The IgG test, however, yielded 11–17% and 15–44% positives in healthy travellers vaccinated against JE and YF, respectively [9]. On the other hand, the ELISA kit detected IgM-antibodies only 4–8 days after the onset of clinical symptoms, since dengue antibodies tend to rise late in the acute phase of a primary infection [19]. Therefore, to increase the sensitivity of an early diagnosis of dengue, it is highly recommended to perform both dengue RT-PCR and dengue E/M-specific capture IgM and IgG ELISA if available [6,8].

Among 1,031 sero-negative patients presenting in the acute phase of illness at our travel clinic, five were tested positive by RT-PCR during the first three days after onset of symptoms. Of the 21 patients presenting during this time period with positive PanBio ELISA results, only one was confirmed by virus isolation but 20 were classified as false positive (i.e. negative confirmatory antibody tests and negative RT-PCR/virus isolation). This finding highlights that clinicians should consider preferably RT-PCR to diagnose dengue-infections in patients seeking medical care up to five days after onset of symptoms, and that ELISA-results on blood collected during this period of disease should be used to demonstrate seroconversion or rising antibody titres in combination with a follow-up sample but not to confirm the diagnosis with a single serum sample. However, it must also be pointed out that a negative RT-PCR test result does not exclude a dengue infection.

Furthermore, a long time period (> 15 days) between the onset of the acute symptoms and the blood collection was also associated with a higher rate of false positive PanBio-ELISAs. In most of these cases the patients did not have symptoms at the time of presentation but asked for a check-up and the identification of a pathogen that might have caused the fever while travelling.

In our study population, 127 serum samples of travellers with antibody titres indicating probable acute or recent dengue infection in the standard PanBio-ELISA were further analyzed and revealed a high rate (42.5%) of false positive results in single specimen analysis. The confirmatory analysis has been performed by using an E/M-specific capture ELISA which has been found to differentiate reliably between JE, YF, dengue, and West Nile virus infection in previous studies [16]. Furthermore, NS1 isotype- and serotype-specific IgM and IgG-ELISAs have been applied that demonstrated a good correlation with dengue virus PRNT [20]. Overall, it must be concluded that in the absence of these more specific assays, paired serum are required to yield the high specificity of the PanBio ELISAs observed in previous studies [13]. The lacking second serum sample can be regarded as the major factor for the low specificity of the assay in our study.

Travelling in South East Asia was significantly associated with a lower risk to reveal a false positive result in the PanBio-ELISAs when compared to other travel destinations. This phenomenon can be explained by the fact that the positive (and negative) predictive value of a diagnostic test is influenced by the prevalence of the disease in a specific geographic region. Regarding dengue virus activity, Southeast Asia is the most seriously affected area worldwide [21], and travellers to this region were shown to be at highest risk when compared to other regions [4,14,22].

The high specificity of the PanBio IgM ELISA in travellers with previous immunizations demonstrated in a recent study [9] leads to the suggestion that in our population other pathogens might have caused cross reactive antibodies in these sick travellers with high but false positive IgM levels. In one of the investigated patient's sera antibodies against JEV were detected and were probably responsible for the false positive result. In 2 of the 54 patients with false positive dengue ELISA an infection with *Plasmodium falciparum *was diagnosed, in 5 patients an intestinal infection with *Giardia lamblia*, and in one patient with *Strongyloides stercoralis*. However, it might be suggested that none of these later mentioned pathogens have the potential to cause cross-reactivities with the PanBio-ELISA. Overall, possible explanations for false positive PanBio-ELISA results include cross-reactive flavivirus-specific IgM antibodies, non-specific bindings of IgM antibodies from other infections, and rheumatoid factor. As mentioned earlier, a major factor for the low positive predictive value of the PanBio-ELISA is the fact that only single serum samples have been tested. This, however, was subject to our study.

Underlining the good validity of the retrospective analysis, clinical data of the patients are very similar to those gained prospectively in a population of 465 travellers with imported dengue infection presenting at 43 European travel clinics [23] where 63% were reported to have headache, 52% muscle pain, and 34% rash. In our study population, rash was the only clinical symptom significantly more frequently observed in patients with confirmed dengue infection when compared to febrile travellers without dengue. However, this symptom was present in only 24% of the patients with dengue infection and was therefore not helpful to increase the positive predictive value of the PanBio-ELISA. In contrast, thrombocytopenia was present in 50% of the dengue patients and supported the correct diagnosis in combination with the single serum analysis, but would hardy serve as a single indicator due to the low prevalence. In a study performed in adults presenting to a hospital with febrile illnesses in Singapore, rash was identified as the most reliable clinical parameter to predict dengue fever [24]. In this population living in a dengue endemic area, the combination of simple laboratory parameter (white cell count, haemoglobin, prothrombin time, creatinine, and bilirubin levels) and rash was demonstrated to potentially predict dengue achieving a sensitivity of 84% and specificity of 85%.

## Conclusion

The examination of paired serum samples remains the most reliable serodiagnostic procedure for dengue. However, if only one blood sample is available, a single positive ELISA-result carries a high rate of false-positivity and should be confirmed using a second and more specific diagnostic technique. In the absence of further testing, platelet and white blood cell counts are useful for the correct interpretation. In our population, the combination of these two features increased the positive predictive value of a PanBio-ELISA test from 50% to 90.5%

In the very early stage of illness only RT-PCR or virus isolation can reliably confirm the diagnosis of dengue. Positive ELISA-results during the first three days of illness should be mistrusted by the investigator and only be used as the baseline titre for a second sample. Especially in cases with only low positive IgM-titres and in the absence of above described blood count alterations a second serum sample collected during the convalescent phase is highly recommended and other potential and treatable reasons for travel-related fever should be excluded in this stage. On the other hand, if a single specimen serological result in combination with the clinical-haematological findings is highly suggestive for dengue infection, further expensive investigations could be omitted.

## Competing interests

The author(s) declare that they have no competing interests.

## Authors' contributions

OW participated in the design and coordination of the study, did the statistical analysis, and prepared the first draft of the manuscript. KS participated in the design of the study and revised the draft manuscript. PYS carried out the ELISA and RT-PCR studies. MN carried out the immunofluorescence assays. CF participated in the statistical analysis and revised the draft manuscript. JHH participated in the serological analyses and contributed to the study with in-depth knowledge of the laboratory diagnosis of dengue infections. TJ participated in its design, and revised the draft manuscript. All authors read and approved the final manuscript.

## Pre-publication history

The pre-publication history for this paper can be accessed here:


